# S100A8 and S100A9 in experimental osteoarthritis

**DOI:** 10.1186/ar2917

**Published:** 2010-01-27

**Authors:** Hala Zreiqat, Daniele Belluoccio, Margaret M Smith, Richard Wilson, Lynn A Rowley, Katie Jones, Yogambha Ramaswamy, Thomas Vogl, Johannes Roth, John F Bateman, Christopher B Little

**Affiliations:** 1 Tissue Engineering and Biomaterials Research Unit, School of AMME J07, Faculty of Engineering, Bosch Institute, University of Sydney, Corner of Shepherd and Cleavland Street, New South Wales 2006, Australia; 2 Murdoch Childrens Research Institute and the Department of Paediatrics, University of Melbourne, Royal Children's Hospital, Flemmington Road, Parkville, Victoria 3052, Australia; 3 Raymond Purves Bone and Joint Research Laboratories, Kolling Institute of Medical Research, University of Sydney at Royal North Shore Hospital, Reserve Road, St. Leonards, New South Wales 2065, Australia; 4 Institute of Immunology, University Hospital of Muenster, Roentgenstrasse 21, D-48149 Muenster, Germany

## Abstract

**Introduction:**

The objective was to evaluate the changes in S100A8 S100A9, and their complex (S100A8/S100A9) in cartilage during the onset of osteoarthritis (OA) as opposed to inflammatory arthritis.

**Methods:**

S100A8 and S100A9 protein localization were determined in antigen-induced inflammatory arthritis in mice, mouse femoral head cartilage explants stimulated with interleukin-1 (IL-1), and in surgically-induced OA in mice. Microarray expression profiling of all S100 proteins in cartilage was evaluated at different times after initiation of degradation in femoral head explant cultures stimulated with IL-1 and surgically-induced OA. The effect of S100A8, S100A9 or the complex on the expression of aggrecan (*Acan*), collagen II (*Col2a1*), disintegrin and metalloproteases with thrombospondin motifs (*Adamts1*, *Adamts 4 *&*Adamts 5*), matrix metalloproteases (*Mmp1*, *Mmp3*, *Mmp13 *&*Mmp14*) and tissue inhibitors of metalloproteinases (*Timp1*, *Timp2 *&*Timp3*), by primary adult ovine articular chondrocytes was determined using real time quantitative reverse transcription polymerase chain reaction (qRT-PCR).

**Results:**

Stimulation with IL-1 increased chondrocyte *S100a8 *and *S100a9 *mRNA and protein levels. There was increased chondrocyte mRNA expression of *S100a8 *and *S100a9 *in early but not late mouse OA. However, loss of the S100A8 staining in chondrocytes occurred as mouse OA progressed, in contrast to the positive reactivity for both S100A8 and S100A9 in chondrocytes in inflammatory arthritis in mice. Homodimeric S100A8 and S100A9, but not the heterodimeric complex, significantly upregulated chondrocyte *Adamts1*, *Adamts4 *and *Adamts 5*, *Mmp1*, *Mmp3 *and *Mmp13 *gene expression, while collagen II and aggrecan mRNAs were significantly decreased.

**Conclusions:**

Chondrocyte derived S100A8 and S100A9 may have a sustained role in cartilage degradation in inflammatory arthritis. In contrast, while these proteins may have a role in initiating early cartilage degradation in OA by upregulating MMPs and aggrecanases, their reduced expression in late stages of OA suggests they do not have an ongoing role in cartilage degradation in this non-inflammatory arthropathy.

## Introduction

S100 proteins are low molecular weight (9 to 14 kDa) intracellular calcium-binding proteins that control key cellular pathways including regulation of the cytoskeleton [1], cell migration and adhesion [2], and host oxidative defense [3,4]. Some S100 proteins have also been demonstrated to have important extracellular pro-inflammatory effects and cytokine-like activities in addition to their intracellular functions. When released from cells, S100A8, S100A9, S100A11, and S100A12 act as unconventional inflammatory cytokines [5,6]. Therefore, not only the expression of these proteins by cells, but also their release into the extracellular environment may have important implications on their activity in a given tissue.

S100A8 and S100A9 are found intracellularly in granulocytes, monocytes, and early differentiation stages of macrophages [7,8]. A clear increase and role for S100A8 and S100A9 in the synovium and macrophages in inflammatory arthritis has been established [9,10]. Extracellular S100A8 is considered a pro-inflammatory molecule because of its effect on cytokine synthesis [11] and upregulation of destructive matrix metalloproteinases (MMP) and disintegrin and metalloproteases with thrombospondin motifs (ADAMTS) enzymes by macrophages [10,12]. In contrast, S100A9 alone was previously shown not to activate phagocytes and, when it forms a complex with S100A8, to decrease the activity of this S100 protein [11]. Chondrocytes have also been shown to express S100A8 and S100A9 [13] and their upregulation following stimulation with IL-1 and oncostatin-M, suggested a possible role in cartilage repair or inflammation-induced degradation [14]. Recently, increased S100A8 and S100A9 staining of chondrocytes in inflammatory arthropathies in mice and humans was reported [9]. This same study also demonstrated that extracellular S100A8 stimulated expression and activity of various matrix-degrading metalloproteinases by a chondrocyte cell line, and aggrecanolysis in mouse patella explant cultures [9]. These results suggested that in inflammatory arthritis, extracellular S100A8 secreted from inflammatory cells or the chondrocytes themselves may be an important mediator of cartilage matrix degradation.

In contrast to the significant role of infiltrating inflammatory cells and synovial pannus in rheumatoid arthritis (RA), cartilage breakdown in osteoarthritis (OA) is driven primarily by the chondrocytes. Although considered to be a non-inflammatory arthropathy, a role for chondrocyte-derived cytokines in maintaining elevated proteolysis of aggrecan and collagen in end-stage human OA cartilage has been demonstrated [15]. To date, however, the changes in S100A8 and S100A9 expression and protein localization and the potential role of these two proteins in cartilage destruction during the onset and progression of OA as opposed to inflammatory arthropathies has not been investigated. Furthermore, although it has been shown that S100A8 can induce catabolic enzymes expression in chondrocyte cell lines [9], no previous studies have established whether S100A8 has a similar effect in primary adult articular chondrocytes or if S100A9 or the S100A8/A9 complex has a similar effect. We investigated the immunolocalization of S100A8 and S100A9 in sections of antigen-induced arthritis (AIA); the effect of IL-1α on *S100a8 *and *S100a9 *expression and immunolocalization in mouse cartilage explants *in vitro*; the *in vivo *expression and immunolocalization of S100A8 and S100A9 in cartilage during progressive cartilage destruction in an OA compared with an inflammatory arthritis model in mice; and the effect of S100A8 and S100A9 on the expression by primary adult ovine articular chondrocytes of key extracellular matrix molecules, matrix degrading enzymes, and their inhibitors.

## Materials and methods

### Mouse osteoarthritis model

All animal experimentation was conducted with approval from the Royal North Shore Hospital Animal Care and Ethics Committee (protocols 0051-005A and 0506-019A). OA was induced in 10-week-old male C57BL6 mice by medial meniscal destabilization (MMD) of the right knee [16]. Joints with no surgery or subjected to sham-operation (exposure of the medial menisco-tibial ligament but no transection) were used as controls. Animals were sacrificed at 2, 4, 8 and 16 weeks after surgery (n = 3 per time point) for histology and immunohistology.

Additional 10 week-old C57BL6 male mice underwent bilateral surgery with the right knee undergoing MMD while the left knee underwent a sham-operation. Animals were sacrificed at one, two, and six weeks after surgery (n = 7 per time point). The joints were dissected to expose the articular cartilage, tibial epiphyses were isolated and placed in RNA later containing 20% EDTA, decalcified at 4°C for 72 hours and then embedded in optimal cutting temperature (OCT) compound and stored at -80°C. Serial 7 μm coronal cryo-sections were fixed in ethanol, air-dried, and non-calcified medial tibial plateau articular cartilage from previously assigned areas of cartilage fibrillation and loss of toluidine blue staining were microdissected and isolated using a Veritas microdissection system (Molecular Devices, Sunnyvale, CA, USA).

### Mouse cartilage isolation and culture

Femoral head cartilage was isolated from 24-day-old C57B6 wild type mice and cultured for two or four days in serum-free medium with or without 10 ng/ml recombinant human IL-Iα (PeproTech, London, UK [17]. In four-day cultures the media was changed after two days. At termination, femoral heads were either stored at -20°C in RNA later (Ambion, Austin TX, USA) prior to RNA extraction or embedded in OCT and stored at -80°C prior to immunostaining.

### Chondrocyte isolation and culture

Chondrocytes from four-year-old ovine knee articular cartilage were isolated by sequential pronase and collagenase digestion and grown to confluence in serum-containing media [18]. Cells were incubated overnight in serum-free medium prior to stimulation for 24 hours with serum-free medium containing 10^-7 ^or 10^-8 ^M recombinant human S100A8, S100A9, or the complex of both (n = 6 replicates/treatment). Recombinant human S100A8, S100A9, or heterocomplex with no contaminating lipopolysaccharise (LPS) were expressed and purified as described [13,19,20].

### RNA extraction

At the termination of culture, ovine chondrocytes were washed with PBS, and then lysed with TRIzol (Invitrogen Life Technologies, Mulgrave, Victoria, Australia). Mouse femoral heads were pulverized using a liquid nitrogen-cooled tissue mill. Pulverised femoral heads and micro-dissected cartilage from frozen sections of sham or MMD-induced OA joints were extracted with TRIzol. Total cellular RNA in TRIzol extracts was isolated from all samples by RNeasy kit (Qiagen, Doncaster, Victoria, Australia) including an on-column DNase I (Qiagen, Doncaster, Victoria, Australia) digestion. RNA was quantified using Sybrgreen (Molecular Probes, USA) with 18S/28S rRNA as a standard (Sigma-Aldrich, Castle Hill, NSW, Australia). Three femoral heads were pooled to generate a representative RNA sample for each *in vitro *treatment and were analysed by microarray expression profiling. Micro-dissected cartilage from three separate joints was pooled to account for biological variability, and provide a representative sample of sham or MMD cartilage RNA at each time point for microarray analysis. The RNA from the remaining four sham and MMD joints were analysed separately using quantitative RT-PCR (qRT-PCR) for *S100a8 *and *S100a9 *to verify the results from the microarray analysis.

### Ovine chondrocyte quantitative reverse transcription polymerase chain reaction

Changes in mRNA expression in cultured primary ovine chondrocytes were quantified using real-time qRT-PCR as previously described [21]. Reverse transcription (RT) reactions were undertaken with 1 μg total RNA (Omniscript RT kit, Qiagen, Doncaster, Victoria, Australia). All samples underwent RT at the same time to avoid potential variations in experimental conditions. Aliquots of cDNA were amplified by PCR using specific ovine primer sets (Table 1). All PCR reactions generated single products with confirmed sequences (SUPAMAC, Sydney University, NSW, Australia). The differentiated phenotype of control cultures of primary ovine chondrocytes in monolayer was confirmed by examining gene expression relative to *Gapdh*. However, all 'housekeeping' genes evaluated (*Gapdh*, *Actb*, *Hprt*, ubiquitin) showed differential regulation by S100 proteins (data not shown). Therefore, to evaluate the changes induced by S100A8, S100A9, or the heterocomplex, gene expression in all cultures including controls was subsequently corrected for total RNA [22] and the effect of added S100 proteins expressed as fold change from control cultures.

**Table 1:** Ovine-specific real time PCR primer pair sequences, annealing temperatures and product size

Target gene	Sequence	Anneal temp (°C)	Product size (bp)	Accession number or reference if published
*Acan*	F - TCA CCA TCC CCT GCT ACT TCA TC R - TCT CCT TGG AAA TGC GGC TC	58	105	[21]
*Adamts1*	F - CCA ACT GGA GCC ACA AAC ATT G R - GGA CAG AGT GAA GTC GCC ATT C	55	126	[GenBank: XM_589626]
*Adamts4*	F - AAC TCG AAG CAA TGC ACT GGT R - TGC CCG AAG CCA TTG TCT A	60	149	[44]
*Adamts5*	F - GCA TTG ACG CAT CCA AAC CC R - CGT GGT AGG TCC AGC AAA CAG TTA C	55	97	[21]
*Col2a1*	F - TGA CCT GAC GCC CAT TCA TC R - TTT CCT GTC TCT GCC TTG ACC C	55	154	[GenBank: X02420]
*Mmp1*	F - CAT TCT ACT GAC ATT GGG GCT CTG R - TGA GTG GGA TTT TGG GAA GGT C	55	122	[GenBank: AF267156]
*Mmp3*	F - TCC CCC AGT TTC CCC TAA TG R - GAT TTC TCC CCT CAG TGT GCT G	58	124	[GenBank: AF135232]
*Mmp13*	F - GGT GAC AGG CAG ACT TGA TGA TAA C R - ATT TGG TCC AGG AGG GAA AGC G	58	349	[21]
*Mmp14*	R - CCC AGT GCT TGT CTC CTT TGA AG	56	126	[GenBank: AF267160]
*Timp1*	F - GGT TCA GTG CCT TGA GAG ATG C R - GGG ATA GAT GAG CAG GGA AAC AC	57	265	[GenBank: S67450]
*Timp2*	F - ACT CTG GCA ACG ACA TCT ACG G R - TCT TCT TCT GGG TGG CAC TCA G	57	261	[GenBank: M32303]
*Timp3*	F - CTT CCT TTG CCC TTC TCT ACC C R - TCT GGT CAA CCC AAG CAT CG	57	286	[GenBank: NM_174473]
*Gapdh*	F - CCT GGA GAA ACC TGC CAA GTA TG R - GGT AGA AGA GTG AGT GTC GCT GTT G	58	139	[GenBank: U94889]

*Acan *= aggrecan; *Adamts *= a disintegrin and metalloproteinase with thrombospondin motifs; F = forward primer; *Gapdh *= glyceraldehyde 3-phosphate dehydrogenase; *Mmp *= matrix metalloproteinase; R = reverse primer; *Timp *= tissue inhibitor of metalloproteinases.

### Mouse cartilage RNA amplification, microarray hybridization and qRT-PCR

To quantify changes in all *S100 *mRNA in cultured mouse femoral heads and micro-dissected tibial cartilage from the OA model, linear amplification in one or two rounds, respectively, was performed using the MessageAmp kit (Ambion, Austin TX, USA) following the manufacturers guidelines. Aminoallyl-modified UTP was incorporated and then labelled with reactive fluorophors Cy3 or Cy5 (GE Healthcare, Rydalmere, NSW, Australia). Duplicate microarrays (Cy3/Cy5 dye-swap with replicate RNA samples) were performed for *in vitro *treatment (i.e. control versus IL-1 at each time point), and sham versus MMD (at one, two and six weeks). Labelled RNA was hybridized to 44 k whole genome oligo microarrays (G4122A, Agilent Technologies, Forest Hill, Victoria, Australia). The arrays were scanned on an Axon 4000B scanner and features extracted with GenePix Pro 4.1 software (Molecular Devices, Sunnyvale, CA, USA). Raw data was processed using a print-tip Loess normalization [23] using limmaGUI software [24]. Mean log2-transformed expression ratios and B-statistic values (log posterior odds ratio [23] were calculated for all direct comparisons [25]. Data is plotted as the average fold-change compared with control for femoral head culture experiments, or average fold-change with MMD compared with sham surgery at each time point. The changes in *S100a8 *and *S100a9 *mRNA expression in micro-dissected mouse tibial cartilage following MMD were validated by qRT-PCR in four separate animals at each time point, and the median fold change in MMD compared with sham-operated joints was calculated. These analyses were performed as previously described [26] using mouse-specific primer pairs (*S100a8 *forward - TGCGATGGTGATAAAAGTGG, reverse - GGCCAGAAGCTCTGCTACTC; *S100a9 *forward - CACAGTTGGCAACCTTTATG, reverse - CAGCTGATTGTCCTGGTTTG), and the expression of *S100a8 *and *S100a9 *were normalized using the geometric mean expression of two housekeeping genes [27], *Atp5b *(forward - GGCTGATAAGCTGGCAGAAG, reverse - GGAGAGATCAGTTGCAGTGCT), and *Rpl10 *(forward - TTGAAGACATGGTTGCTGAGA, reverse - AGGACCACGATTGGGGATA). These two housekeeper genes were shown by microarray expression profiling to be unchanged during the onset and progression of OA in the MMD mouse model (data not shown).

### Immunolocalization of S100A8 and S100A9

Sections from archival paraffin blocks of male C57BL6 mouse knee joints with either AIA (7 and 28 days after induction) or saline injection from a previous study [16] were prepared at the same time as serial sections from the mouse knee joints with surgically-induced OA. Together with frozen sections from femoral head cultures, slides were immunolocalized with polyclonal antibodies to S100A8 and S100A9 (generously provided by Professor Caroline Geczy [13] and Dr. Thomas Vogl [9,12]). Immunostaining with the two different S100A8 and S100A9 antibodies gave similar results, and therefore only those obtained using the antibodies supplied by Zreiqat and colleagues [13] are shown. Negative controls included omitting the primary antibody or using an equivalent concentration of rabbit immunoglobulin (Ig)G as a control for nonspecific antibody binding. Images representative of typical immunostaining in mouse knee joints with either OA or AIA are presented. The antibodies to S100A8 did not recognize recombinant S100A9 on western blotting and vice versa (data not shown); the anti-S100A8 and anti-S100A9 polyclonal antibodies did not cross-react with human S100A12, S100B or S100A1 [28,29]. The specificity of immunostaining was further validated by pre-absorption with 10 nmol of the recombinant proteins for one hour at room temperature prior to immunolocalization.

### Statistical analysis

Comparisons of parametric data were undertaken using the unpaired Student's t-test with Benjamini-Hochberg correction for multiple comparisons [30]. Differential expression in microarray analysis was assumed for B-statistic of 1.0 or more [23].

## Results

### S100A8 and S100A9 immunolocalization in antigen induced arthritis

**Figure 1:**
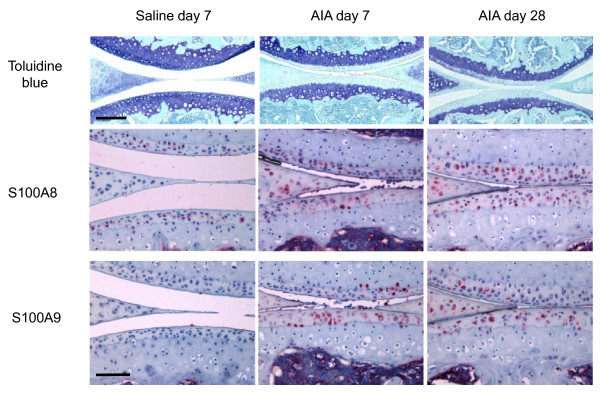
**Toluidine blue/fast green and S100A8 and S100A9 immunostained paraffin sections of medial femoro-tibial compartments of mouse knee joints from saline-injected compared with AIA at days 7 and 28**. Scale bar = 100 μm. AIA = antigen-induced arthritis.

As previously described [16] there is a complete loss of proteoglycan staining in the non-calcified cartilage by seven days post AIA induction (Figure 1). Chondrocytes, particularly in the deep and to a lesser extent the superficial zone of the non-calcified articular cartilage were positive for S100A8, but not S100A9 in control (saline-injected) joints. S100A8 reactivity remained positive in the non-calcified cartilage in AIA joints, either at levels similar to or increased compared with saline-injected (non-inflamed) control joints (Figure 1). This positive chondrocyte S100A8 staining was observed up to 28 days after induction of AIA even though there is significant resolution of the synovial inflammation at this time point [16]. Chondrocytes in the non-calcified articular cartilage became immunopositive for S100A9 at seven days after AIA induction, and remained positive at 28 days (Figure 1). Meniscal fibrochondrocytes showed positive S100A8 and S100A9 immunostaining in AIA joints at all times. Cells in the bone marrow of all joints and inflammatory cells in the synovium (not shown) and joint space (Figure 1, day seven) were also strongly positive for S100A8 and S100A9.

### Immunolocalization of S100A8 and S100A9 in mouse OA

**Figure 2:**
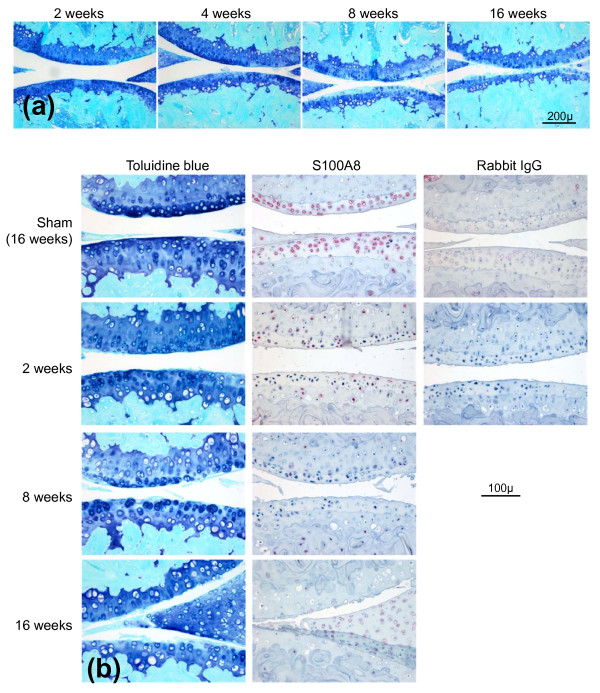
**Histopathological examination of osteoarthritic changes in mouse knee (femoro-tibial) joints following medial meniscal destabilization (MMD)**. **(a) **Progressive cartilage damage at 2, 4, 8 and 16 weeks following medial meniscal destabilization-induced osteoarthritis (OA) in mouse knee joints. Toluidine blue/fast green stained paraffin sections. Scale bar = 200 μm. **(b) **Serial sections stained with toluidine blue or with S100A8 immunolocalization in mouse knees at different times following medial meniscal destabilization (MMD) or sham surgery (at 16 weeks). Scale bar = 100 μm. Negative control sections were immunostained using an equivalent concentration of rabbit IgG.

Meniscal destabilization induced a progressive deterioration of the articular cartilage in the medial femoro-tibial joint (Figure 2a) with no evidence of synovial inflammation as previously described [16]. Cartilage damage at two weeks was characterized by a focal loss of proteoglycan from the non-calcified cartilage in the central weight-bearing region of the tibial plateau but no structural damage. The area of proteoglycan loss expanded with time and at eight weeks was accompanied by evidence of surface fibrillation and some areas of erosion. By 16 weeks there was full thickness erosion of non-calcified cartilage to cover over 50% of the joint surface.

**Figure 3:**
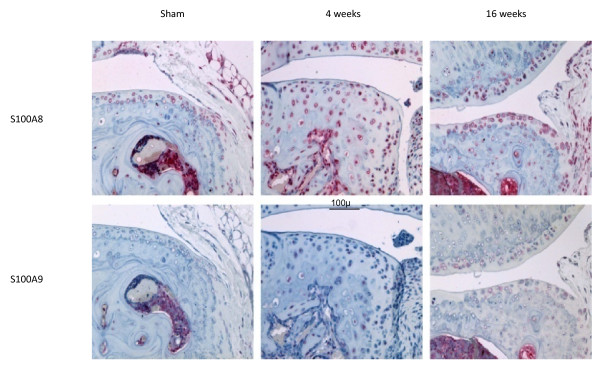
**S100A8 and S100A9 immunostaining in cartilage and subchondral bone at the joint margins following sham operation or medial meniscal destabilization at 4 and 16 weeks**. Scale bar = 100 μm.

There was no difference in immunostaining for S100A8 and S100A9 between non-operated and sham-operated joints at any time (results not shown) and therefore only sham-operated results are included (Figure 2b). As described in saline-injected joints above, chondrocytes throughout the non-calcified cartilage in non-operated and sham-operated joints showed positive staining for S100A8 (Figure 2b) but not S100A9 (not shown). In marked contrast to the AIA model, with induction of OA there was a loss of chondrocyte S100A8 immunoreactivity in the non-calcified cartilage compared with the corresponding sham-operated joint (Figure 2b). The loss of S100A8 chondrocyte staining extended beyond the area of proteoglycan loss defined by decreased toluidine blue staining. Even at late stages of OA with extensive cartilage erosion, chondrocytes in the remaining intact cartilage in the load-bearing region of the joint had reduced or lost S100A8 reactivity (Figure 2b, week 8 and 16). However, S100A8 reactivity was still apparent in chondrocytes at the joint margins at all time points, and in the calcified cartilage, bone marrow and bone of developing and mature osteophytes (Figure 3). In contrast, chondrocytes showed little positive S100A9 immunostaining in marginal regions in either normal (non-operated or sham-operated) or OA joints, although bone marrow and some osteocytes were positive (Figure 3).

### Temporal changes in *in vivo *expression of S100 genes in mouse OA

**Figure 4:**
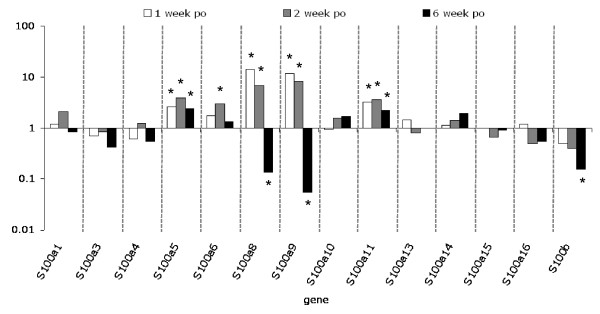
**Fold change in *S100 *gene expression measured by microarray expression profiling, in micro-dissected cartilage from surgically-induced OA compared with sham-operated joints 1, 2 and 6 weeks post-operatively**. Pooled samples were used from three sham or medial meniscal destabilization (MMD) joints per time point. *B-statistic ≥ 1.0. OA = osteoarthritis; po = post-operatively.

To determine whether the loss of S100A8 immunostaining in cartilage in OA was due to decreased expression, microarray mRNA expression profiling of the S100 protein family was performed [see Additional file 1]. The expression of all S100 genes in chondrocytes in the non-calcified articular cartilage at one, two and six weeks post-induction of OA was determined and results expressed as fold change compared with the sham-operated joints (Figure 4). The expression of a number of *S100 *mRNAs including *S100a5, S100a6, S100a8, S100a9, S100a11*, and *S100b *was significantly (B-statistic ≥ 1.0) regulated in chondrocytes following surgical induction of OA. The most highly regulated were *S100a8 *and *S100a9*, and, unlike other *S100 *family members, they showed differential regulation with 7- to 14-fold upregulation in early (week one and two) stages of OA and a 7- to 18-fold decrease compared with sham-operated levels in late-stage (week six) disease. The change in *S100a8 *and *S100a9 *expression measured using qRT-PCR showed some variability between the individual animals. Nevertheless, three of the four animals at each time point showed the same direction (i.e. increase or decrease) of change, and the median fold change (n = 4) had a similar temporal pattern to that observed in microarray analysis: 3.9- and 11-fold increase in *S100a8 *and *S100a9*, respectively, in early OA (two weeks), and a 16- and 25-fold decrease, respectively, in late-stage (six weeks) MMD-induced OA.

### *In vitro *regulation of S100A8 and S100A9 in mouse cartilage

**Figure 5:**
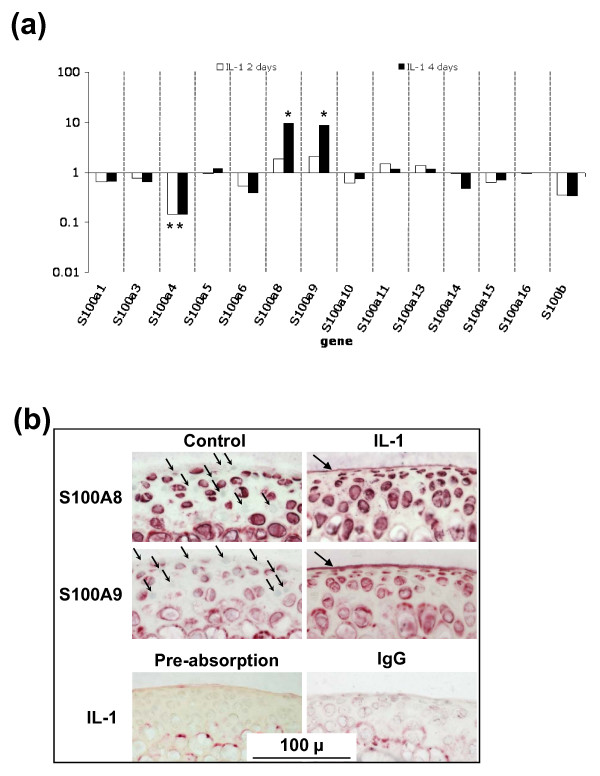
**Changes in S100 proteins in cultured mouse articular cartilage**. **(a) **Fold change in *S100 *gene expression, measured by microarray expression profiling, in mouse femoral head cartilage cultured with or without IL-1 for two or four days compared with control cultures measured by microarray expression profiling (pooled sample from three femoral heads/treatment/time). *B-statistic ≥ 1.0. **(b) **S100A8 and S100A9 immunostaining in frozen sections of mouse femoral head articular cartilage following four days of culture with IL-1. Substantial numbers of chondrocytes did not stain with S100A8 and S100A9 in the control cultures (small arrows). In IL-1-stimulated cultures the intensity and proportion of positively stained chondrocytes increased. In addition, the surface matrix lamina stained positive for both S100A8 and S100A9 (large arrows). Negative controls included sections of IL-1-stimulated cartilage incubated with equal concentration of IgG, or localized with anti-S100A8 following pre-absorption with recombinant S100A8 protein. Scale bar = 100 μm.

In light of the distinct temporal change of *S100 *mRNAs, and particularly *S100a8 *and *S100a9*, in chondrocytes in OA, we investigated the regulation of expression of this family of proteins during IL-1-induced degradation in cartilage explants using microarray expression profiling [see Additional file 2]. In contrast to the changes seen in OA (Figure 4), chondrocyte expression of *S100a5, S100a6*, and *S100a11 *was not regulated by IL-1 *in vitro *(Figure 5a). *S100a8 *and *S100a9 *were both upregulated by IL-1 at day four (10 and 9 fold, respectively) but not day two, while expression of *S100a4 *was decreased by IL-1 (about seven fold) at both two and four days (Figure 5a). At day 2, *S100a8 *and *S100a9 *protein was localized to the chondrocytes in control cultures, although not all cells were positive (Figure 5b). In comparison to the loss of S100A8 immunostaining seen in surgically-induced OA (Figure 2b), the number and/or intensity of chondrocyte S100A8 and S100A9 staining was increased in IL-1-stimulated cartilage at day four, particularly in flattened surface zone cells, such that all cells as well as the surface matrix lamina were positively immunostained for both proteins (Figure 5b). Weak staining of calcified cartilage matrix but not chondrocytes or the surface matrix was evident with equivalent concentrations of rabbit IgG even in IL-1-stimulated cultures, and staining was abolished pre-absorption with the recombinant protein (Figure 5b).

### S100A8 and S100A9, but not S100A8/S100A9 complexes regulate chondrocyte gene expression

**Figure 6:**
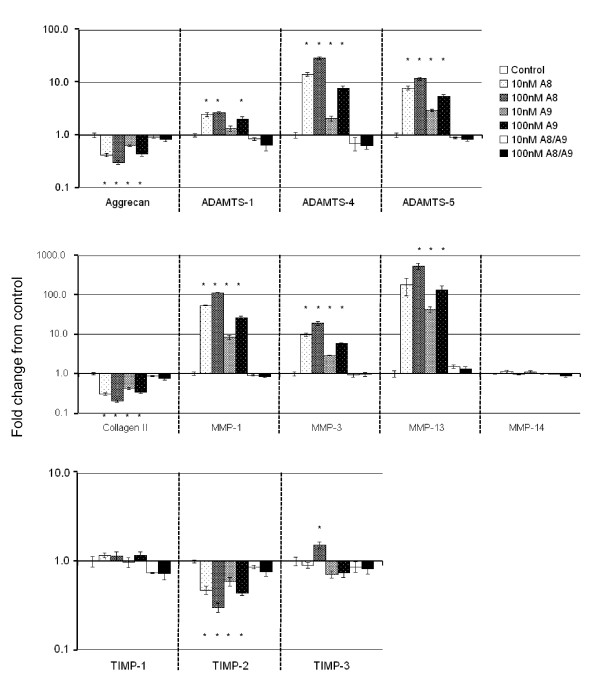
**Fold change (± SEM) compared with unstimulated cultures in gene expression measured by real-time qRT-PCR in primary ovine chondrocytes cultured for 24 hours in monolayer with 10 or 100 nM S100A8, S100A9 or S100A8/S100A9 complex**. n = 6 per culture condition. * = significant difference compared with control cultures (*P *< 0.027).

We sought to determine whether S100A8, S100A9, and/or their heterodimeric complex had a similar pro-catabolic effect in primary chondrocytes as previously reported for S100A8 alone in a chondrocyte cell line [9]. We therefore compared mRNA expression of key cartilage matrix proteins, enzymes, and their inhibitors in primary adult articular chondrocytes stimulated with physiologically-relevant concentrations of S100A8, S100A9, or their complex. Primary ovine chondrocytes in monolayer culture maintained their anabolic chondrocyte phenotype with relatively high expression relative to *Gapdh *of aggrecan (3.0 ± 0.6), type II collagen (3.0 ± 0.4), tissue inhibitors of metalloproteinases *(Timp)1 *(1.4 ± 0.4), *Timp2 *(2.3 ± 0.3), and *Timp3 *(1.4 ± 0.3) and generally low catabolic enzyme expression (*Adamts1 *= 0.9 ± 0.1; *Adamts4 *= 0.2 ± 0.1; *Adamts5 *= 0.4 ± 0.1; *Mmp1 *= 0.06 ± 0.01, *Mmp3 *= 0.3 ± 0.1; *Mmp13 *= 0.06 ± 0.03 and *Mmp14 *= 1.8 ± 0.06). None of the genes examined were significantly regulated by the heterodimeric S100A8/S100A9 complex (Figure 6). In contrast, both S100A8 and S100A9 homodimers caused a dose-dependent downregulation of chondrocyte aggrecan expression whereas *Adamts1*, *Adamts4*, and *Adamts*5 mRNA levels were all significantly increased (Figure 6). Collagen type II mRNA was significantly decreased by S100A8 or S100A9, while *Mmp1, Mmp3*, and *Mmp13 *were dose-dependently upregulated. *Timp1 *and *Timp3 *mRNAs were largely unchanged, while *Timp2 *mRNA levels were decreased by S100A8 and S100A9 (Figure 6). The pattern of most highly upregulated genes by S100A8 and S100A9 (10^-7^M) were similar with, from most to least upregulated, *Mmp13 *then *Mmp1 *then *Adamts4 *then *Mmp3 *and finally *Adamts5*.

## Discussion

In this study S100A8 was immunolocalized in chondrocytes in normal murine articular cartilage *in vivo*, and for the first time we showed that this intracellular S100A8 is lost in OA. This contrasted sharply with the retention or increase of S100A8 immunostaining in chondrocytes in cartilage from inflammatory arthropathies such as AIA. S100A9 protein was not detectable in chondrocytes in the normal non-calcified region of the articular cartilage, and although it increased in inflammatory arthritis, no chondrocyte or cartilage immunostaining was detected in OA. We found that the lack of S100A8 and S100A9 protein localization in chondrocytes early in OA, was not associated with a decrease but rather a significant increase in mRNA expression for both proteins. Although increased chondrocyte mRNA for *S100a8 *and *S100a9 *could be induced by IL-1 *in vitro*, this was associated with an increase in cell and cartilage matrix staining for the two proteins. Together with the distinctly different chondrocyte expression profile of other S100 proteins in IL-1-stimulated compared with OA murine cartilage, this suggests that the early upregulation of S100A8 and S100A9 in surgically-induced OA was not due to increased IL-1 activity. Importantly, increased mRNA levels for both *S100a8 *and *S100a9 *in early OA was associated with a loss of cellular staining, suggesting that these S100 proteins may be secreted from the cells and act as extracellular signaling molecules. We have now shown that homodimeric S100A9 promotes increased catabolic enzyme and decreased matrix protein gene expression in chondrocytes very similar to that induced by the S100A8 homodimer. In contrast, the heterodimeric complex failed to alter chondrocyte metabolism, suggesting that a dysregulation in expression and/or secretion of the two subunits may play a significant role in their potential bioactivity.

Taken together the above novel findings suggest that the regulation of *S100a8 *and *S100a9 *expression and secretion from chondrocytes could play a role in the early stages of cartilage degradation in OA, and highlight the significant differences in the pathogenesis of cartilage destruction in OA versus inflammatory joint diseases. The strong positive chondrocyte staining for both S100A8 and S100A9 observed in AIA in the current study was in accord with previously reported results in this inflammatory arthritis model [9]. However, we found that normal mouse articular chondrocytes were positive for S100A8, the specificity of which was confirmed by the lack of staining with equivalent pre-immune IgG and pre-absorption of the antibody with recombinant S100A8. This positive S100A8 staining contrasts with a previously reported lack of immunostaining in normal mouse knees [9], but is consistent with positive S100A8 and S100A9 staining in murine and human growth plate chondrocytes [13], and non-stimulated H4 murine chondrocyte cells [9]. The reason for this discrepancy is unclear but may relate to differences in staining sensitivity due to fixation, decalcification, antibodies, and/or immunostaining methods used. Indeed, in frozen sections of mouse femoral head cartilage we could show positive S100A9 as well as S100A8 staining. This different staining pattern in the femoral head cartilage compared with adult joints, may be due to the age of the mice from which the cartilage was obtained, and/or that the tissue was cultured for four days prior to immunostaining. Nevertheless, the results are consistent with active synthesis of both S100A8 and S100A9 proteins by chondrocytes in normal non-calcified articular cartilage. The change in S100A8 immunostaining in surgically-induced mouse OA was restricted to the load-bearing areas of articular cartilage, whereas localization in other tissues and at the joint margins was unaltered. This differed in AIA where increased meniscal staining for S100A8 and S100A9 was observed in association with positive articular chondrocyte staining. This suggests that local factors such as mechanical overloading of the cartilage, rather than humoral agents affecting the whole joint such as cytokines or growth factors, play a significant role in regulating the metabolism of these proteins in cartilage in OA.

MMP-2 and MMP-9 have been shown to degrade S100A8 and S100A9 [31] and both of these MMPs are upregulated in OA cartilage [32,33] and could potentially explain the loss of immunostaining in the mouse model. It is also possible that the increased chondrocyte *S100a8 *and *S100a9 *mRNA in early MMD-induced OA was not translated into protein, through micro-RNA silencing pathways predicted to act on the mRNA of both genes [34,35]. However, we speculate that the loss of chondrocyte immunostaining for both S100A8 and S100A9 in early OA while mRNA expression for both proteins is increased, may be due to their secretion from the cell. S100A8 and S100A9 are released from macrophages and neutrophils during inflammation [6], and this secretion is concomitant with loss of cellular immunostaining [36], similar to that observed in the chondrocytes in the present study. S100A9 is released from IL-1-stimulated mouse cartilage *in vitro*, while S100A8 is not detected in this same conditioned media, suggesting either differential release or extracellular processing/degradation of the two proteins [37]. In the current study there was evidence of extracellular release of S100A9, and to a lesser extent S100A8, with positive immunostaining in the surface matrix lamina of IL-1-stimulated mouse femoral head cartilage (Figure 5b). However, the chondrocytes still remained strongly immunopositive for both S100A8 and S100A9 in this IL-1-stimulated cartilage despite secretion of the S100 proteins, which contrasts with lack of chondrocyte staining in OA mouse joints. To date, we have not been able to confirm if there is increased soluble S100A8 or S100A9 in articular cartilage in OA. It remains unclear, therefore, whether release of S100A9 and/or S100A8 from chondrocytes occurs in early OA or with excessive mechanical loading of cartilage.

Release of S100A8 and S100A9 proteins from chondrocytes into the extracellular space, would facilitate their activity as cytokine-like molecules in early OA. We showed that exogenous/extracellular S100A8, and for the first time S100A9 homodimer, could have a role in initiating cartilage degradation by decreasing chondrocyte expression of aggrecan (*Acan*) and collagen II (*Col2a1*), but increasing *Adamts1*, *Adamts4*, *Adamts5*, *Mmp1, Mmp3*, and *Mmp13 *mRNA levels. The increase in metalloproteinase mRNA was not balanced by a similar increase in TIMPs, promoting a potential imbalance in enzyme/inhibitor ratios and matrix degradation once the pro-MMPs are activated. This is consistent with the recent report showing increased aggrecanolysis in murine patella explant cultures stimulated with S100A8 [9]. The effects of S100A8 on primary ovine articular chondrocytes were in general agreement with that reported in the synovium and macrophages [12], and the H4 murine chondrocyte cell line [9], although some subtle differences were noted. We found no regulation of *Mmp14 *by S100A8 in chondrocytes, whereas this enzyme was upregulated in synovium [12]. It has been suggested that the chondroprotection in inflammatory arthritis in S100A9 knock-out mice could be due to the concomitant lack of S100A8 in these animals [9]. Our results have now shown that S100A9 homodimer itself could play a role in cartilage breakdown by inducing similar regulation of potential cartilage-degrading enzymes in chondrocytes as S100A8.

Thus far there is no explanation as to why the heterodimer is inactive and the homodimers are active in chondrocytes. We speculate that the heterodimers may require a trigger for activation in contrast to the homodimers, which are constitutively active. For the murine heterodimer one such trigger is LPS, and activation of cells by LPS is amplified in the presence of the murine heterodimer [11]. It has been suggested that S100A8/S100A9 complex formation results in conformational change and altered biological function of the individual proteins [11,38]. Oligomerization of the heterodimer with calcium/zinc binding may result in steric masking of the receptor-binding epitope [38]. This is consistent with the fact that S100A8/S100A9 complex failed to regulate gene expression in chondrocytes. Previously, however, the heterodimercomplex but neither homodimer was found to be active in stimulating endothelial cells [39], and the complex upregulated MMP13 in macrophages to a similar level as the S100A8 homodimer [12]. These divergent results suggest that the S100 proteins may elicit distinct effects in different cell types within the joint. It would be interesting in the future to determine whether these differential effects are driven by variation in expression of potential receptors for these S100 proteins, such as cell-surface heparan sulfate proteoglycans [40] or toll-like receptor-4 (TLR4) [11], which are expressed by chondrocytes [41,42]. TLR4 in particular has been strongly implicated in joint inflammation and cartilage destruction in experimental inflammatory arthropathies in mice [43].

## Conclusions

We have shown that extracellular S100A8 and S100A9 homodimers can both stimulate a degradative response in articular chondrocytes. Dysregulation of the synthesis and release of these two proteins by chondrocytes could play a role in cartilage destruction in arthritis. However, our studies comparing surgically-induced OA with AIA in mice, suggests that although S100A8 and S100A9 may have a role in initiating early cartilage degradation in both arthropathies, they are unlikely to have a significant role in the ongoing cartilage degradation in chronic/late-stage OA. Defining the differences in pathophysiological pathways and mechanisms in different arthritic conditions and in different stages of disease is important in designing better therapies.

## Abbreviations

ADAMTS: disintegrin and metalloproteases with thrombospondin motifs; AIA: antigen-induced-arthritis; Ig: immunoglobulin; IL: interleukin; LPS: lipopolysaccharide; MMD: medial meniscal destabilization; MMPs: matrix metalloproteinases; OA: osteoarthritis; PBS: phosphate-buffered saline; qRT-PCR: quantitative reverse transcription polymerase chain reaction; RA: rheumatoid arthritis; RT: reverse transcription; TIMPs: tissue inhibitors of metalloproteinases.

## Competing interests

The authors declare that they have no competing interests.

## Authors' contributions

HZ contributed to study design, acquisition of data, analysis and interpretation of data, and manuscript preparation. DB contributed to acquisition of data, analysis and interpretation of data, and manuscript preparation. MMS contributed to acquisition of data, analysis and interpretation of data, manuscript preparation, and statistical analysis. RW contributed to acquisition of data. LAR contributed to acquisition of data, and manuscript preparation. KJ contributed to analysis and interpretation of data. YR contributed to analysis and interpretation of data, and manuscript preparation. TV contributed to analysis and interpretation of data, and manuscript preparation. JR contributed to manuscript preparation. JFB contributed to manuscript preparation. CBL contributed to study design, acquisition of data, analysis and interpretation of data, manuscript preparation, animal management, and animal surgery. All authors read and approved the final manuscript.

## Supplementary Material

Additional file 1 Excel file containing the raw microarray data from the Agilent 44 K arrays comparing the expression of S100 proteins in micro-dissected non-calcified articular cartilage pooled from three separate animals one, two and six weeks after medial meniscal destabilization or sham surgery. Click here for file

Additional file 2 Excel file containing the raw microarray data from the Agilent 44 K arrays comparing the expression of S100 proteins in femoral head cartilage pooled from three separate cultures under control and IL-1-stimulated conditions for two and four day. Click here for file
